# Employees' experiences of personal and collective work-identity in the context of an organizational change

**DOI:** 10.3389/fpsyg.2025.1382271

**Published:** 2025-03-12

**Authors:** Ola Nordhall, Julia Hörvallius, Mathilda Nedelius, Igor Knez

**Affiliations:** Department of Occupational Health, Psychology and Sports Science, University of Gävle, Gävle, Sweden

**Keywords:** personal and collective work-identity, emotion, cognition, organizational change, theory-driven thematic analysis

## Abstract

In the present study we investigated employees' experiences of personal and collective work-identity in the context of an organizational change. Data consisted of semi-structured interviews with employees that will be affected by the change. We conducted a theory-driven thematic analysis based on four predetermined main themes: personal and collective emotional, and cognitive work-identity. Respondents experienced distinct and unambiguous proudness, bonding, familiarity and missing in their *personal emotional* work-identity, and quite distinct and unambiguous coherence, reflection, mental time travel, but ambivalent, or even lack of, correspondence in their *personal cognitive* work-identity. They experienced a mix of distinct and ambiguous organizational proudness, esteem and affective commitment in their *collective emotional* work-identity. They experienced distinct and unambiguous identification with the organization, but ambivalent assimilation and incorporation of the organization in their *collective cognitive* work-identity. Such a complexity in the employees' work-identity experiences also indicates complexity in their organizational change reactions.

## Background

To maintain competitiveness in an ever-changing and dynamic world, it is required that organizations adapt to conditions which may significantly affect them (Sendrea, 2017). One such important strategy is organizational change (Liang et al., 2022, see Oreg et al., 2013 for an overview) involving employees' partial or total adaptation to a concept, idea or behavior within the organization (Odor, 2018).

Most organizational changes are implemented in a top-down manner (Schulz-Knappe et al., 2019). This may be met with resistance, especially by those who appraise the change as a threat to the organization's core identity or to their own organizational identity (Fugate et al., 2012; van Dick et al., 2016). Resistance to organizational change may also occur due to uncertainties related to an individual, groups and/or the organization. However, organizational change may also be appraised as a positive challenge. This may trigger general positive change orientations, such as a strong sense of confidence, eagerness and hopefulness (see Fugate et al., 2012 for an overview).

One factor of importance in understanding employees' reactions to organizational change is their work-identity (Fugate and Kinicki, 2008; Liang et al., 2022; Yue, 2021). That is, how individuals define and categorize themselves in terms of individual and social attributes in relation to their occupational work, i.e., who they are at work (Ashforth and Mael, 1989; Hogg, 2012; Knez, 2016; Nordhall et al., 2018). Work-identity implies all kinds of meanings that the individual has connected with his/her own self, based on group memberships, social roles and/or individual unique characteristics (Kragt and Day, 2015).

Personal work-identity denotes individual identification with the professional work, while a collective work-identity refers to identification with work groups or the organization itself (Knez, 2016; Nordhall et al., 2018). Both identities include emotional and cognitive bonds in terms of work-related thoughts, feelings, memories and reasoning (Knez, 2014, 2016; Nordhall et al., 2021). Previous studies have shown personal- and collective work-identity to differently associate with a wide range of work- and organizational behaviors, norms and attitudes (see Riketta, 2005; Riketta and Van Dick, 2005; Knez, 2016; Lee et al., 2015 for overviews). The emotional and cognitive components of personal- and collective work-identity may differently account for some of these associations (see Nordhall and Knez, 2018; Nordhall et al., 2018; Nordhall, 2021). Below we will outline this in more detail.

### Personal and collective work-identity

Work-identity occurs at individual and social levels (Knez, 2016; Xenikou, 2014) referring to the personal (I/Me) and the collective (We/Us) work-related identifications (Millward and Haslam, 2013; Knez, 2016).

Personal work-identity is accounted for by an autobiographical memory perspective (Klein, 2014; Klein and Nichols, 2012) asserting that the individual self, by our memories, is a narrative product of its past and vice versa (Knez et al., 2017; Knez and Nordhall, 2017). Additionally, autobiographical memories create a sense of self-continuity over time, such as, “I was therefore I am” (Bluck and Liao, 2013, p. 7). Personal work-identity has the personal work/career as foci of distinguishing oneself from others (Brewer and Gardner, 1996) “in order to preserve the personal self, the personal story and its memories” (Knez, 2016, p. 3).

Collective work-identity implies “the individual's knowledge that he [or she] belongs to certain social groups together with some emotional and value significance to him [or her] of his [or her] group membership” (Tajfel, 1972, p. 292). Such a group membership implies an emotional attachment to the group/organization by cognitive depersonalizations of the individual self (Tajfel and Turner, 1986; see Hogg, 2012 for an overview).

According to the social identity perspective (e.g., Ashforth and Mael, 1989), including theories of social identity (Tajfel and Turner, 1986) and self-categorization (Turner et al., 1987), individuals define themselves in terms of membership of social categories, thereby attributing prototypes of such social categories to themselves. Accordingly, individuals perceive themselves in terms of the characteristics they share with other in-group members (Haslam and Ellemers, 2011; van Knippenberg and Van Schie, 2000). Collective work-identity emanates from the need of social belonging, “in order to be part of the collective self, the collective story and its memories” (Knez, 2016, p. 3) and implies perception of oneness with e.g., a work organization (Ashforth and Mael, 1989; van Knippenberg and Sleebos, 2006).

#### Emotion and cognition in work-identity

Personal and collective work-identities comprise basic psychological processes of feeling and thinking about oneself in connection to one's work (Brown, 2017; Johnson et al., 2012; Klein et al., 2004) grouped into components of emotion and cognition (Knez, 2016; Xenikou, 2014, see also van Dick and Wagner, 2002).

In *personal* work-identity, the *emotion* component includes processes of affective *familiarity, proudness, bonding, missing* and *emotional agency* (work as a part of oneself) between an employee and his/her occupational work. The *cognition* component of *personal* work-identity includes processes of *coherence* (continuity in the self-work relation across time), *correspondence* (adaptive interactions between the self and its working contexts), *reflection* (upon work-related memories), *mental time travel* to the workplace and *cognitive agency* (work-related thoughts and memories as part of oneself) (Knez, 2016; Nordhall et al., 2020; see also Knez, 2014). In the present study we investigated all these processes except for agency (emotional and cognitive) which was not included in the interview guide due to its complicated nature.

In *collective* work-identity, the *emotion* component includes processes of *esteem* (feelings of being complimented if someone were to speak well of the organization), *proudness* and *affective commitment* (feelings of personal insult if the organization is slandered). The *cognition* component of *collective* work-identity involves definitions and self-categorizations of the employee and includes processes of e.g., *identification* (perception of the organization as “we” rather than ”they/them”), *assimilation* (of organizational success as one's own success) and *incorporation* (of other people's perception of the organization) (Nordhall and Knez, 2018; Nordhall et al., 2020, see also Ashforth and Mael, 1989; Mael and Ashforth, 1992; Mael and Tetrick, 1992; van Knippenberg and Sleebos, 2006). In the present study we investigated all these processes in collective work-identity.

Work-identity as an important psychological phenomena as related to organizational change reactions will be described below.

### Organizational change and work-identity

Individuals with stronger personal work-identity may show resistance to organizational change because it may generate a break-up of a previously established work-identity by forcing them to establish a new one (van Dick et al., 2006; van Vuuren et al., 2010). The individual employee may fear a loss of personal control. Change may furthermore be perceived as a threat to different loyalties and the “ways of thinking” at work, generating stress in individuals (Ashforth, 2001; Jetten et al., 2002; Nordhall and Knez, 2018; see Knez, 2016 for an overview). It has been suggested that stronger personal cognitive work-identity may constitute a mental effort that may heighten responses to stressful events at work such as an organizational change. Consequently, such work-related thinking may generate mental health problems associated with an organizational change (see Nordhall et al., 2018; Nordhall, 2021 for an overview).

However, individuals with a stronger personal work-identity may also have a stronger sense of employability. That is, a psychological construct fostering pro-active adaptability toward work and career. Employability may also facilitate a positive change-orientation by reducing uncertainties associated with organizational change. Individuals with a stronger personal work-identity may proactively pursue identity-consistent interests and anticipate future organizational change in terms of challenge appraisals, and thus exhibit a positive change-orientation (Fugate and Kinicki, 2008). Individuals with stronger emotional bonding in the personal work-identity may additionally have stronger intrinsic work motivation and better mental health. This might constitute psychological resources facilitating positive change-orientations toward an organizational change (Knez, 2016; Nordhall and Knez, 2018; Nordhall et al., 2018).

From a social identity perspective, Jetten et al. (2002) showed that stronger work-group identity in contrast to stronger organizational identity associates with more negative feelings about an upcoming change.

Furthermore, individuals with a strong organizational affiliation may develop emotional bonding to the organization in terms of attachment/belongingness/closeness (Knez, 2016). The stronger an individual identifies with the organization, the stronger will his/her values, norms, acceptance and loyalty to the organization be. This may facilitate employees' acceptance of organizational decisions of e.g., change (Johnson and Jackson, 2009; Johnson et al., 2006; Mael and Ashforth, 1992). Individuals with a strong sense of cognitive oneness with the organization perceive oneself and the work organization as ”we.” Perception of the organization's successes as one's own successes implies that one feels personally affected if the organization is doing well or not (Ashforth and Mael, 1989). Employees are also more likely to exhibit stronger cooperative behavior in organizational change if they perceive the organization's goals as their own goals (Knez, 2016; van Dick et al., 2018). Also, Yue (2021) showed that for employees with weaker organizational identification, leaders' charismatic rhetoric to address the immediate change is of greater importance.

In contrast, van Dick et al. (2016) reported that employees with a strong organizational identity may exhibit resistance to organizational change if the change is perceived as a threat to the organization's identity. The employees may indirectly perceive this as a threat to their own identity, especially if the individual has stronger emotional bonding to the organization (Nordhall et al., 2018, 2020). Additionally, stronger emotional bonding to the organization can make the individual's self-esteem and reputation dependent on the organizational success *per se*. This relates to the concept of organization-based self-esteem, suggesting that the employees' self-esteem is connected to their organizational attachment (Pierce and Gardner, 2004). This may make employees more vulnerable to stress-related factors such as an organizational change.

The present study investigated a business area within a company that will be separated from the remaining company group and form its own limited company. This organizational change will mean a new management system, a new market label and a new company name.

### Aim

Emotional- and cognitive processes in work-identity have shown to differently account for associations between personal- and collective work-identity, respectively, and work-and health related outcomes (see Knez, 2016; Nordhall, 2021). However, this has not been consistently applied in a context of organizational change. Such application may shed light on experiences of specific work-related processes during organizational change.

Relationships between work-identity and organization change reactions have to a large extent been investigated from a social identity perspective focusing organizational i.e., collective work-identity (see Hogg, 2012 for an overview) with a quantitative approach as a frame of reference. Even though qualitative experiences of work-identity in the context of an organizational change have been investigated to some extent, a vast majority of such studies have used an inductive, theory generating, approach (see Drzensky and van Dick, 2013; Oreg et al., 2013; van Dick et al., 2018 for overviews). This contrasts with the present study that used a deductive, theory-driven, qualitative approach where the interview guide and analyses were based on theoretical predetermined themes (see Azungah, 2018).

Given this, the *aim* of the present study was to investigate employees' experiences of personal and collective work-identity in a context of an organizational change.

More precisely, how may employees within a business of manufacturing stainless steels experience their emotional and cognitive bonding of personal- and collective work-identity in the context of organizational change?

Accordingly, individual experiences are investigated by deductive themes derived from well-supported occupational theories of personal- (Knez, 2016) and collective work-identity (Ashforth and Mael, 1989; Turner et al., 1987). The theoretically defined themes of emotional and cognitive personal- and collective work-identity are thus given an experiential ideographic content by the participants' individual accounts. This facilitates the understanding of these types of work-identity experiences in the context of organizational change. Also, hereby the conceptualization and relation of these organizational phenomena are elaborated.

Finally, no *direct* data on the organizational change *per se* were investigated. Respondents' answers on the work-identity questions were *intertwined* with the context of an organizational change, see below.

## Methods

The context of the present study implies that a business area will be separated from the remaining company group and form its own limited company, including new: management system, market label, and company name. Given that these changes will be implemented in a near future, the present study involves the phenomena of pre-change *organization* and *work identities* (see Gleibs et al., 2008; van Dijk and van Dick, 2009; van Dick et al., 2018).

A thematic, theory-driven method based on semi-structured interviews was chosen to investigate respondents' experiences of personal and collective work-identity in the context of the above-mentioned organizational change (Knez, 2016; Nordhall et al., 2020; Xenikou, 2014). This deductive analysis was based on an autobiographical memory perspective of personal work-identity (Knez, 2014, 2016), and a social identity (Mael and Ashforth, 1992; Riketta, 2005) and self-categorization (Turner et al., 1987) perspective on collective work-identity. In accordance with previous research (Knez, 2016; Knez and Nordhall, 2017; Nordhall et al., 2021), four pre-determined main themes were applied: (1) *personal emotional* work-identity; (2) *personal cognitive* work-identity; (3) *collective emotional* work-identity; and (4) *collective cognitive* work-identity. Each main theme included three/four psychological processes (see Nordhall et al., 2021) i.e., sub-themes that were reformulated into questions, defining the semi-structured interview guide; see below.

### Participants

The study consisted of a total of seven participants. By this number of participants, we obtained richness of information in that the participants were of different ages and genders and had different organizational positions and employment times, see below. Also, for the 6th and 7th interview the content of the answers were similar to the other participants' answers in some respects. Hence, it is reasonable that saturation was obtained by these interviews (see Hennick et al., 2017, for a critical discussion of saturation in terms of number of participants, see also O'Reilly and Parker, 2013; Thorne, 2020). The participants included four white- and three blue-collar workers. The white-collar workers were desk officials (market service managers, flow managers, system technician) and the blue-collar workers were operative industrial workers (system- and production operators). The participants, working within a business of manufacturing stainless steels in the middle part of Sweden, were employees in the business area of the company that in the near future will undergo an organizational change. All participants (white- and blue-collar) were supposed to experience the near future organizational change within the same time frame i.e., the organizational change was supposed to be implemented at the same time for all employees that would be affected by the organizational change. Three of the participants were women and four men. Mean age was 51 years (SD 9.12), range 39–61 years. Mean employment time within the organization was 20 years (SD 9.80), range 5–39 years.

### Materials

In the present study, the four predetermined themes were investigated by main and follow-up questions (see Appendix 1 for the interview guide). The questions were asked in relation to the background information, about the near future organizational change and its implications, given to all participants at the start of each interview (see Procedure section below). Hence, the participants' experiences of work-identity were expressed in the context of an organizational change, which in this case was characterized as a “pre-change” work-identity. This refers to the self-work bonding just before the implementation of an organizational change, while “post-change” work-identity refers to the self-work bonding that occurs shortly after the organizational change has been implemented (Jetten et al., 2002; van Dijk and van Dick, 2009; van Dick et al., 2018).

#### Personal emotional and cognitive work-identity

The questions of personal emotional and cognitive work-identity were based on the questionnaire, “Work-related Self Measure,” developed by Knez (2016; see also Knez et al., 2019; Nordhall and Knez, 2018). This scale contains items of emotional and cognitive processes of personal work-identity (Nordhall et al., 2021). In the present study some of these were reformulated into interview questions. The questions in the interview guide captured experiences of personal *emotional* work-identity in terms of the following work-identity processes: affective *proudness, bonding/familiarity* and *missing* between an employee and his/her occupational work. Questions of personal *cognitive* work-identity captured the processes of *coherence* (continuity in the self-work relation across time), *correspondence* (adaptive interactions between the self and its working contexts), *reflections* upon work-related memories and *mental time travel* to the workplace (Knez, 2016; Nordhall et al., 2021; see also Knez, 2014).

#### Collective emotional and cognitive work-identity

The questions of collective emotional and cognitive work-identity were based on Mael and Ashforth's (1992) questionnaire, “Identification with a Psychological Group Scale” (see also Ashforth and Mael, 1989; Mael and Ashforth, 1992). Nordhall and Knez (2018) have developed this scale into a division of emotion and cognition components of collective work-identity (see also Nordhall et al., 2020). In the present study some of these items were reformulated into interview questions. The questions in the interview guide captured experiences of collective *emotional* work-identity in terms of organizational *esteem* (feelings of being complimented if someone were to speak well of the organization), *proudness* of organizational belonging and *affective commitment* (feelings of personal insult if the organization is slandered). Questions of collective *cognitive* work-identity captured processes of *identification* (perception of oneness with the organization), *assimilation* (of organizational success as one's own success) and *incorporation* (of other people's perceptions of the organization).

Data of the present study may be made available by the corresponding author on request.

### Procedure

We contacted an HR Manager of the organization who asked employees to participate in the present study. After approval from eight employees, the HR Manager sent their contact information to us by email. The employees then received a covering letter about participation in the study. All eight employees approved participation in the study. Due to the Covid-19 situation, restrictions regarding external visitors to the organization were implemented at this time. Accordingly, the planned face-to-face interviews were scheduled as telephone interviews. All participants were informed about this, which they approved. Due to changes in workload one of the participants refrained from participating.

At the time of the interviews, two of the authors of the present study conducted the interviews and the participants were verbally informed about the purpose and ethical considerations of the study and estimated time for the interview. To ensure that all respondents' answers were based on the same basic knowledge about the near future organizational change, they all received the same information about the change and its implications at the start of the interviews. The participants were asked if they consented to the interview being recorded and were given an opportunity to ask questions about the study's content and structure. The participants gave their informed consent to participate prior to the interview. They were also informed that completion of the interview was taken as an indication of their informed consent to participate in the present study.

One semi-structured interview was conducted with each participant. The interviews were conducted on speakerphone and all interviews were recorded with an Ipad and a cellphone. We conducted the interviews in a separate room to reduce the risk of disturbances. Participants were also asked if they wanted to be in a meeting room at the organization for the interview. The duration of the interviews varied between 22 and 46 min.

### Data analysis

We conducted a deductive, theory-driven, thematic analysis in line with the Hayes (1997, see also Hayes, 2000) model involving four steps (for a similar approach, see Azungah, 2018). First, the study's four theoretical predetermined main themes, including the sub-themes (psychological processes) were formulated. Second, data were prepared in the form of transcripts. All seven interviews were transcribed in their entirety. Third, we analyzed each theme separately and determined which data belonged to each theme. The transcripts were coded by identifying key concepts as initial coding categories in accordance with the predetermined main- and subthemes. The process was repeated once again to ensure that all relevant data were properly coded and sorted under the correct theme. Fourth, we read the text for each theme separately and analyzed the respondents' answers to detect any underlying meanings. In conclusion we summarized all descriptions under each theme regardless of who said what; then we selected quotes from the transcripts that were considered to reflect adequately the summaries for each theme.

### Research ethics

According to Swedish juridical restrictions of research ethics (see Etikprövningsmyndigheten/The Ethics Review Authority, n.d.) no formal ethical approval is needed for the type of research conducted in the present study. This because no sensitive personal data were collected, such as the health of the participants. However, the present study followed the research ethic principles of the Declaration of Helsinki (see World Medical Association, n.d.) and APA (American Psychological Association, 2020, n.d.) regarding informed consent from the participants, treatment of participants and handling of research data and results. All participants gave their informed consent regarding participation, participation was voluntary, the participants had the right to cancel their participation at any time, preferably during the course of the study with regard to the consent requirement, and they were anonymous with reference to confidentiality requirements. All participants were informed that their individual answers could not be linked to a specific individual. Finally, all participants were asked if they agreed to the interviews being recorded, and they also received information about the purpose of the recordings.

### Trustworthiness in the present study

Criteria of trustworthiness have been met by credibility, dependability, and transferability (see Graneheim and Lundman, 2004; Rapp et al., 2021).

#### Credibility

Organizational change might be experienced in different ways by desk officials and operative industrial workers. Accordingly, by including these two types of employees i.e., both white and blue collar workers we got different work role perspectives and experiences which is of relevance in order to obtain richness of information of work-identity and organizational change (see Knez, 2016; Morgan and Zeffane, 2003; Rashid et al., 2004). Additionally, participants of different ages, genders and employment times within the present organization were included in the present study. This further contributed to a greater variation in experiences of the current phenomena, thereby strengthen the credibility of the present study.

Dependability was obtained by high level of agreement between the researchers of the present study concerning all steps of the data analysis. Furthermore, we asked the same interview questions, and an open dialog between the researchers contributed to consistent judgements and interpretations during the data collection and analyses.

Transferability was obtained by consistently applying two well supported theoretical frameworks of autobiographical memory and social identity. As well as, by providing the background for organizational change and its implications, detailed descriptions of the pre-determined themes (and sub-themes), quotes from respondents for each sub-theme and by demonstrating that the respondents' individual experiences of the work-identity processes (i.e., sub-themes) are in line with the theoretical accounts of these processes.

## Results

In the result section below, the respondents' answers are presented in accordance with the study's four predetermined main themes of personal and collective emotional, and cognitive work-identity, implying a pre-change identification with the occupational work and work organization (see Table 1 for a summary of the main concepts that emerged in participants' answers). To maintain the participants' anonymity, the respondents are referred to as R1-7. The names of the organization and business area were erased from the answers.

**Table 1:** Main experiences that emerged in participants' answers as related to the deductive main themes (bold) and sub-themes (italics) of type of identity (personal/collective) by type of bond (emotional/cognitive) in a context of organizational change.

	**Personal work-identity**	**Collective work-identity**
**Emotional**	*Proudness*: feeling confident and competent; long-term occupational service; duty; expectations. *Bonding & Familiarity*: job satisfaction; cooperation and unity with co-workers; task variation; being needed. *Missing*: co-workers; personal control; safety; development; routines.	*Esteem*: feeling happy; not a personal compliment *Proudness* & *Affective commitment*: take offense; not a personal insult; equivalent to slandering a family member; anxious to know the arguments in support of the slandering.
**Cognitive**	*Coherence*: way of thinking and acting; time spent at work; trust and responsibility. *Correspondence*: no distinct connection between occupational work and current life; work abilities of value in private life. *Reflection & Mental time travel*: social issues at work; improvement for co-workers; work-related thinking; working from home during illness.	*Identification*: the organization as “we”; strongly included in the organization. *Assimilation*: positive spirit and calmer working conditions and atmosphere in times of organizational success; organizational success does not affect one personally. *Incorporation*: organization's reputation as important from a market perspective.

### Personal emotional work-identity

Several respondents reported that they experienced a distinct and unambiguous occupational *proudness* in their personal emotional work-identity. Some of them described this in terms of feeling confident and competent in their work role, and that this was a result long-term service within the occupation. As one of the respondents put it: “Yes, I am quite competent and have a lot of experience and want to do a good job. So yes, I feel pride in what I do here” (R 2). However, some of the respondents expressed hesitancy regarding their occupational *proudness*; in terms of, “doing one's duty” or “do what one is expected to do” or “do the best one can,” rather than an unambiguous proudness in their occupational work.

Regarding experiences of affective *bonding* and *familiarity*, several of the participants reported that the meaning of work was to have fun and job satisfaction. According to several respondents, work-related *bonding* was due to cooperation and unity with co-workers. Experiences of task variation and being needed in the work tasks were expressed as important in work-related *bonding* and *familiarity*. One of the respondents said: “I get to do different things and feel satisfied with my job because it is quite varied. I also feel that it is nice to feel needed” (R 7). By this, bonding and familiarity are in some ways dependent on social aspects of the work situation.

Several respondents expressed feelings of *missing* their job when not at the workplace, especially in terms of missing one's co-workers, for example: “Most of the time one misses one's co-workers, after all, they are the ones you miss the most (R 4).” Some respondents expressed the *missing* in terms of personal control, safety, development and work as creating routines, such as: “I'm somehow a part of it, that's what I may miss when I'm not working (R 2).” As with bonding and familiarity, for some employees, experiences of missing were dependent on social aspects of the work situation.

### Personal cognitive work-identity

Regarding *coherence* in personal cognitive work-identity, some of the respondents expressed that they have a close relation to their pre-change occupational work, and that their occupational work has affected who they are today, how they think and act. Several respondents also said that this is due to long-term work experiences and how much time they spend at work: “A great part of oneself is your job given that you spend so much time of your life at work” (R 1). Furthermore, this type of self-work relation was experienced as result of work-related trust and responsibility, as expressed by R5: “I am not the same person as before you are given responsibility, trust if you are responsible. It is precisely this trust that makes you grow and gain even more responsibility. So that you change as a person and not only as an employee.”

By this, in terms of coherence some respondents experienced a quite distinct cognitive bonding in their personal work-identity.

Regarding the *correspondence*, most of the respondents did not experience a distinct connection between their occupational work and their current private life. However, some said that their occupational work has given them abilities that may be of some value in their private life. For example, R2 said that at work he/she “may have some exercise in handling people and their different ways of being, which I can use in my private life.”

Several of the participants spoke of distinct experiences of *reflection* and *mental time travel*, in that they reflect upon their occupational work when not at work. Reflection took the form of pondering upon social issues at work and how to improve the situation for co-workers:

“I can say that my brain may not disconnect from work for long, no. No, I think a lot about different work things. How things can get better in the group and… yes, improvements then. It often goes round and round in my head.” (R 3)

*Reflection* and *mental time travel* were also expressed in terms of thinking about work-related tasks and duties, and in terms of doing work despite being off sick at home. Some participants thought that this type of *reflection* and *mental time* might be a mental challenge one has to deal with.

### Collective emotional work-identity

Regarding organizational *esteem* in collective emotional work-identity, several respondents explained that they would be happy and feel good about themselves if someone were to speak well of the organization. One of the respondents said: “Yes, but then you get a little proud and think that they think it is a good company. It can make you a little happy and positive about it too” (R 7). However, most of the respondents declared that they would not perceive praise of the organization as a personal compliment. One respondent expressed a mixed experience if someone were to speak well of the organization: “Well, I don't know if I feel something special then, but maybe it warms my heart a little.” (R 3). By this, they did not express a distinct but rather an ambiguous or ambivalent emotional bonding in their collective work-identity.

Regarding organizational *proudness* and *affective commitment*, several of the respondents expressed that they would probably take offense if the organization were being slandered. Several respondents explained that they would probably not perceive it as a personal insult, but that this was due to the context of the slandering. However, one said that s/he would perceive slandering of the organization as a personal insult and compared it with slandering a family member:

I get sad or angry. No, I kind of don't like it. Somehow, it is like when someone is slandering a family member or the one you love. [] I always defend the organization in some way, because it is an important part of my life. It is much like my own family, I do not speak ill of it either. And I get very annoyed when people are speaking ill about our company. (R 2)

If someone were to slander the organization, some respondents also explained that they would be anxious to know the arguments in support of the claim. One respondent said that his/her reaction to such a statement would depend on whether it is justified:

If they say something that I do not agree with, I can give a greater insight into it than those who do not work there probably would do. Do they say something like that (the organization) seems to be the best workplace in the world, yes, yes, it can be but I may also present circumstances that makes it less good to work for the company. (R 1)

One of the respondents who expressed that s/he wanted to experience proudness of the workplace explained that s/he thought it would be difficult if the media portrayed the organization in a bad way:

It wouldn't be that nice to say that you work for a company that has gained a bad reputation today if anyone asks, because then people who have watched the news would connect it with a scandal as well. So I can say that this is very important. (R 6)

Accordingly, in terms of proudness and affective commitment, some of the respondents did express a distinct- while some expressed an ambiguous or ambivalent emotional bonding in their collective work-identity.

### Collective cognitive work-identity

Regarding the respondents' *identification* with the organization as part of their collective cognitive work-identity, they all expressed that they speak in terms of “we” when talking about the organization. The respondents explained that this is because they work there and therefore experience a “we-feeling.” One respondent explained that s/he perceives him/herself as strongly included in it, as: “… it must be us together” (R 3). Several other respondents explained why they talked about the organization in terms of “we,” for example: “I work at (the organization), then I think I am included in it” (R 1). Another respondent (R 2) said:

Yes, but this is a bit of a family affair. It's a big company but still I feel oneness with the company in that I spend a lot of time here and have a lot of commitment, so then it becomes a “we.” I probably have no better explanation, but I work here, it's my business. So, it's ”we.”

By this and in terms of identification the respondents expressed a distinct cognitive bonding in their collective work-identity.

Regarding the respondents' *assimilation* (of organizational success as one's own success), most said that in times of organizational success both working conditions and the atmosphere in the workplace become calmer, more fun and are characterized by a more positive spirit. None of the respondents said that organizational success affected them personally.

The respondents' *incorporation* (of other people's perception of the organization) indicated that the organization's positive reputation was important to them, first and foremost from a market perspective. One (R 6) said that the organization's reputation is important externally because s/he wants to experience pride in his/her workplace:

Yes, you are proud that you can say that you work at (the organization), and people usually have a positive opinion about the organization. Many people become interested; “What do you do there? The company is so big.”

By this, in terms of assimilation and incorporation most of the respondents expressed a somewhat ambiguous cognitive bonding in their collective work-identity.

## Discussion

Knowledge of employees' work-identity experiences may generate better understanding of attitudinal and behavioral reactions to organizational change, since the concept of work-identity encompasses one's self-concept, who one is, and that this might be challenged by an organizational change (Knez, 2016; Nordhall et al., 2021; van Dick et al., 2016, 2018).

As far as we know, previous research has to a large extent investigated relationships between work-identity and organizational change using a quantitative approach and a social identity perspective. When a qualitative approach has been used, it has been applied in terms of inductive methods without a theory-driven base (see Drzensky and van Dick, 2013; van Dick et al., 2018 for overviews).

This contrasts the goals of the present study, to investigate employees' experiences of personal and collective emotional and cognitive work-identity in the context of an organizational change by a deductive, theory-driven approach. Based on well-defined theoretical frameworks four predetermined main themes of personal and collective emotional, and cognitive work-identity were applied (see Ashforth and Mael, 1989; Knez, 2016; Nordhall et al., 2021; Tajfel and Turner, 1986; Turner et al., 1987). See Table 1 for details and result summary.

### Personal work-identity and organizational change

The results indicate that participants had some unambiguous experiences of *personal emotional* work-identity, in terms of distinct but different experiences of *proudness, bonding, familiarity and missing*. These emotional bonds to their personal work implied that the participants felt confident, competent and needed, and that they experienced control, safety and routines in their occupational work control.

The above suggests that participants, with reference to their *personal emotional* work-identity, may not experience the near future organizational change as a threat, and therefore it may not meet it with resistance. This because the participants expressed a distinct meaning of work and job satisfaction, which may foster a sense of employability by reducing the uncertainty of organizational change that, in turn, may facilitate a positive change-orientation (Fugate and Kinicki, 2008). Accordingly, such types of distinct and unambiguous emotional bonding in the personal work-identity may also indicate stronger intrinsic work motivation and better mental health. This may constitute psychological job-resources fostering positive change-orientations toward an organizational change (Knez, 2016; Nordhall and Knez, 2018; Nordhall et al., 2018).

Regarding *personal cognitive* work-identity, some of the participants expressed *coherence* in their person-work bonding in terms of long-term employment affecting who they are today. Also, several expressed distinct experiences of *reflection* upon work and *mental time travel* to work. These cognitive bonds to their personal work were expressed in terms of a close relation between participants and their occupational work and how they are thinking, e.g., pondering upon work-related issues when not at work. However, with reference to *correspondence* most of the participants did not experience a distinct connection between their occupational work and their current private life.

Some of the experiences of personal cognitive work-identity were expressed in terms of mental efforts (reflection and mental time travel). This is in line with previous findings showing stronger personal cognitive work-identity as related to stronger experiences of psychological job demands increasing employees' exhaustion problems (Nordhall et al., 2018, 2020). Additionally, mental efforts in terms of work-related thinking in the person-work bonding may heighten responses to stressful events at work, such as an organizational change (Nordhall, 2021). Given this, for these employees, organizational change may be perceived as a threat to the personal work-identity and the types of loyalties and “way of thinking” that such identification entails, generating stress in employees (Ashforth, 2001; Jetten et al., 2002; Nordhall and Knez, 2018; see Knez, 2016 for an overview).

### Collective work-identity and organizational change

Regarding *collective emotiona*l work-identity, several respondents expressed organizational *esteem*, some would however not perceive praise of the organization as a personal compliment. Several respondents did express organizational *proudness* and *affective commitment*. However, some did not perceive slandering of the organization as a personal insult. For some participants this indicates a distinct collective emotional work-identity while other respondents indicate an ambiguous or ambivalent emotional bonding in their collective work-identity.

Regarding *collective cognitive* work-identity, several respondents expressed that organizational success and other people's perception of the organization may be important to them. However, this was not articulated in terms of a distinct *assimilation* (organizational success as one's own success) and an *incorporation* (other people's perception of the organization) but in terms of positive consequences for the co-workers and the company from a market perspective. Respondents chiefly said that external work-related aspects, and not their collective self-work bonding, are affected when the organization is doing well. This may indicate an ambiguous cognitive collective work-identity (see Nordhall et al., 2021; Wright, 2009).

These results suggest that some of the employees may not perceive the near future organizational change as a major threat to their work-related identity (van Dijk and van Dick, 2009). This because they did not express an unambiguous or distinct collective emotional work-identity, neither distinct assimilation nor incorporation in the collective cognitive work-identity. This may indicate an initiated emotional and cognitive detachment from the organization that is about to undergo an organizational change. Due to this, these employees may experience weak resistance and strong acceptance toward the organizational change. Individuals expressing an ambiguous or a weak organizational identity are less likely to perceive organizational change as threatening the core identity of the organization or their own organizational identity. If they do not perceive the organizational change as a threat, they will probably not establish resistance to the change as a result of their organizational identity (van Dick et al., 2016).

However, in the present study some participants did express a distinct emotional bonding to the work organization Also, all of the participants expressed unambiguous and distinct *identification* i.e., cognitive bonding with the organization in that they easily speak in terms of “we” when talking about the organization. In view of this, those who identify strongly with an organization may show greater readiness for, and acceptance of, organizational change, under the premise that the employee considers the change to be beneficial to the organization (Drzensky and van Dick, 2013). However, unambiguous and distinct identification with the work organization may also make these employees perceive the organizational change as confusing and threatening to their collective self-concept (Ellemers, 2003; Jetten et al., 2002).

Furthermore, employees easily speaking in terms of “we” when talking about the pre-changed organization may have a strong “us vs. them” orientation, thereby perceiving the organizational change as a threat to their in-group identity (Drzensky and van Dick, 2013; Gleibs et al., 2008). In other words, they may perceive the near future separation of the business area as a threat to their own organizational identity or the identity of the organization. That is, they may show strong negative emotions related to the separation of the business area and show resistance to the change (Drzensky and van Dick, 2013; van Dijk and van Dick, 2009). In view of this, a mixed method study including interviews by van Dijk and van Dick (2009) has indicated that employees who do not share salient social category memberships with change leaders, that is a cognitive identification with the organization, experience “dictatorial” leadership style, a lack of opportunities to feedback change ideas; and change leaders' disinterest in receiving staff suggestions about the changes. This is to some extent in line with Nordhall et al. (2021) indicating that collective work-identity cognitions (perception of oneness with the organization) predict organizational emotions (e.g., emotions of change resistance).

### Practical implications

The following practical implication of the present study may be of importance for both employers and employees: Employees may have a strong identification with the organization but do not understand the purpose of the organizational change. Then they will most likely not perceive the change as beneficial for the organization. This may lead to a lower level of readiness and acceptance (Oreg and van Dam, 2009). Organizational changes may be more successful if management considers and manages the potential impacts of the change with stronger organizational identity. This especially if such type of work-identity is shared among the members of the collective (van Dick et al., 2018). Here, manages' implementations of organizational change would benefit from e.g., individual and/or focus-group interviews with employees regarding their emotional and cognitive bonding to their personal work and to the organization prior an organizational change. Not to consider personal- and collective work-identity experiences of the employees prior an organizational change may imply a misjudgment of the attitudes to the organizational change i.e., experiences of (in-)justice of the change. This in turn may lead to a mismatch between the purpose and communication of the organizational change from the employer and the subsequent behavior of the employees that are affected by the change. This may have organizational- and monetary consequences for the employer. It has namely been shown that individuals with stronger collective identity are more disappointed and even consider revenge when treated unfairly, which might be the case for employees that strongly opposes an organizational change (see Cremer, 2006; Knez, 2016; Nordhall and Knez, 2018).

### Limitations

Finally, some limitations of the present study should be mentioned: First, the qualitative approach of the present study with seven participants limits the possibilities to generalize the results to a wider population. Therefore, there is a need to quantitatively investigate the phenomena of the present study using a randomized sample technique and a sample big enough to provide sufficient power to the study. Also, one may use a bigger sample in order to draw more elaborated conclusions based on qualitative data such as the present study. Second, we only interviewed one sample from one company which might limit the generalizability of the results obtained. For future studies it would be of value to interview participants from different organizations regarding their work-identity experiences in the light of similar organizational changes. Also, it would be of value to considering organizational culture, leadership, gender distribution and hierarchical levels within and across companies as explanatory factors. Third, we interviewed the participants prior to the organizational change, thereby only capturing their pre-change work-identity. It would be of value also to interview the same individuals during and after the implementation of the organizational change.

## Conclusions

Using a deductive, theory-driven qualitative approach, the present study adds new knowledge to the understanding of employees' personal and collective work-identity in the context of an organizational change by investigating experiences of emotional and cognitive work-identity processes on both a personal- and a collective work-identity level. By this, the present study moves beyond previous inductive interview studies on work-identity and organizational change. These studies have mainly focused on collective work-identity in terms of organizational membership as related to change leadership. In general, the present results have not been indicated by previous inductive qualitative and/or quantitative studies.

Overall, we have shown: (1) ambivalent and ambiguous, and (2) distinct and unambiguous, emotional and cognitive bonding in respondents' personal and collective pre-change work-identity. This suggests, in practice, that employers should take such a complexity in employees' self-work bonding into consideration when communicating the purpose of an organizational change. This, to constructively handle diverse reactions, such as resistance and submissive acceptance to organizational change.

## Data Availability

The raw data supporting the conclusions of this article will be made available by the authors, without undue reservation.
