# Supplementary material for: Employees' experiences of personal and collective work-identity in the context of an organizational change
Source: Front Psychol. 2025 Mar 12;16:1382271. doi: 10.3389/fpsyg.2025.1382271 (PMC11948747; doi:10.3389/fpsyg.2025.1382271)
Supplement: Supplementary file 1 [file Table_1.docx]

**Appendix 1.**

**Interview guide**

**Background questions**

-Can you tell us about yourself?

- How old are you?

-Have you worked all your life at [name of the organization]?

- For how long have you been working in the production?

-What is your position? What are your duties at work?

-How long have you worked at the organization? How did you get here?

**Background information about the organizational change**

The context of the present study implies that a business area will be separated from the remaining company group and form its own limited company, including new: management system, market label, and company name.

**What is your opinion of the upcoming organizational change?**

**Personal work-identity**

**Personal Emotional Work-Identity**

-*Proudness*: Do you experience professional pride in your work? Why/why not? How would you describe your professional pride/lack of professional pride?

-*Bonding/Familiarity*: What does your work mean to you? How would you describe the “bond” between you and your work?

-*Missing*: Do you miss your work when you are not at work? Why-why not? How do you experience your missing/lack of missing?

**Personal Cognitive Work-identity**

*-Coherence*: Has your work influenced who you are today? Why/in what way?/why not?

*-Correspondence*: Do you feel that there is a connection between your private and

working life? Why/In what way?/Why not?

*-Reflection/Mental Time*: Do you ever reflect on your work when you are not there? Why/in what way do you reflect on it?)/why not?

**Collective Work-Identity**

**Collective Emotional Work-Identity**

*-Esteem*: How do you feel when others speak well of the organization?

Do you think it affects you personally? In what way/why not?

*-Proudness/ Affective Commitment*: How do you feel when others speak badly of the organization? Do you think it affects you personally? In what way/why not?

**Collective Cognitive Work-Identity**

*-Identification*: When you talk about the organization, do you usually say ‘‘we’’ or ‘‘they’’? Why?/Why not?

-*Assimilation*: How do you feel when the organization is doing well? How does it affect you?

-*Incorporation*: Do you feel that the organization’s reputation is important to you? Why do you think so/why don’t you think so?
